# Impact of genetic and non-genetic factors on hepatic CYP2C9 expression and activity in Hungarian subjects

**DOI:** 10.1038/s41598-021-96590-3

**Published:** 2021-08-24

**Authors:** Ferenc Fekete, Katalin Mangó, Máté Déri, Evelyn Incze, Annamária Minus, Katalin Monostory

**Affiliations:** Institute of Enzymology, Research Centre for Natural Sciences, Magyar tudósok 2, Budapest, 1117 Hungary

**Keywords:** Health care, Medical research, Molecular medicine

## Abstract

CYP2C9, one of the most abundant hepatic cytochrome P450 enzymes, is involved in metabolism of 15–20% of clinically important drugs (warfarin, sulfonylureas, phenytoin, non-steroid anti-inflammatory drugs). To avoid adverse events and/or impaired drug-response, *CYP2C9* pharmacogenetic testing is recommended. The impact of *CYP2C9* polymorphic alleles (CYP2C9*2, CYP2C9*3) and phenoconverting non-genetic factors on CYP2C9 function and expression was investigated in liver tissues from Caucasian subjects (N = 164). The presence of CYP2C9*3 allele was associated with CYP2C9 functional impairment, and CYP2C9*2 influenced tolbutamide 4′-hydroxylase activity only in subjects with two polymorphic alleles, whereas the contribution of CYP2C8*3 was not confirmed. In addition to *CYP2C9* genetic polymorphisms, non-genetic factors (co-medication with CYP2C9-specific inhibitors/inducers and non-specific factors including amoxicillin + clavulanic acid therapy or chronic alcohol consumption) contributed to the prediction of hepatic CYP2C9 activity; however, a CYP2C9 genotype–phenotype mismatch still existed in 32.6% of the subjects. Substantial variability in CYP2C9 mRNA levels, irrespective of *CYP2C9* genotype, was demonstrated; however, CYP2C9 induction and non-specific non-genetic factors potentially resulting in liver injury appeared to modify CYP2C9 expression. In conclusion, complex implementation of *CYP2C9* genotype and non-genetic factors for the most accurate estimation of hepatic CYP2C9 activity may improve efficiency and safety of medication with CYP2C9 substrate drugs in clinical practice.

## Introduction

Human CYP2C9 is one of the most abundant drug metabolizing cytochrome P450 (CYP) enzymes, being expressed in the liver at protein level similar to the CYP3A4^1^. CYP2C9 is involved in the metabolism of approximately 15–20% of clinically used drugs, such as anticoagulants (*S*-acenocoumarol, *S*-warfarin), antiepileptics (phenytoin, valproate), non-steroid anti-inflammatory drugs (diclofenac, ibuprofen, flurbiprofen, celecoxib, valdecoxib), oral sulfonylurea antidiabetics (tolbutamide, glyburide), diuretics (torsemide, sulfinpyrazone), and antihypertensive angiotensin II receptor blockers (losartan, irbesartan)^2–4^. Many of these drugs, most prominently warfarin, acenocoumarol, sulfonylureas, valproate and phenytoin have a narrow therapeutic index; therefore, continuous monitoring of blood coagulation, serum glucose level and/or drug concentration is recommended. Substantial inter-individual variability in the metabolism of these drugs has been reported, which is mainly attributed to the genetic polymorphisms of *CYP2C9*^4,5^. More than 60 *CYP2C9* allelic variants have been identified, several of which are associated with changes, typically decreasing, in enzyme activity (https://www.pharmvar.org/gene/CYP2C9, access date: 19 May 2021). The polymorphic CYP2C9*2 and CYP2C9*3 alleles occur at the highest frequencies though with significant interethnic differences^4,6^. In Caucasian populations, CYP2C9*2 is common (10–17%), whereas it is relatively rare in Africans and Asians (2–4.6%), particularly in East Asian populations (0.03%). CYP2C9*3 allele is also frequent in Caucasian and Asian populations (6–7% and 2–11%, respectively), while it is less prevalent in African or African-American ethnicity groups (1%). Other *CYP2C9* allelic variants associated with altered expression or enzyme function are rare (< 0.1%) or absent in Caucasian individuals; though some of them (CYP2C9*5, *6, *8 and *11) occur predominantly in populations with African descent^4^.

CYP2C9*2, one of the well-characterized *CYP2C9* allele, is associated with an amino acid change (Arg144 $\to$ Cys144) and decreased enzyme activity due to the 3608C > T transition in exon 3 (rs1799853). The Arg144Cys amino acid substitution has been demonstrated to affect the interaction of the enzyme with NADPH-cytochrome P450 oxidoreductase that can significantly impair the CYP2C9 catalytic function^7^. However, alterations in the CYP catalytic cycle have also been suggested to diminish metabolic activity of CYP2C9*2^8^. CYP2C9*3 allele with a nucleotide change of 42614A > C in exon 7 (rs1057910) results in an amino acid substitution of Ile359 $\to$ Leu359 in the active site of the enzyme that has been suggested to play an important role in substrate recognition or in formation of iron-oxene complex^8,9^. The Ile359Leu amino acid change leads to a significant decrease in catalytic activity due to an increase in K_m_ values and a decrease in maximal enzyme activity (v_max_) for most CYP2C9 substrates^10,11^. In addition, some single nucleotide polymorphisms (SNPs) in the promoter region of *CYP2C9* have been identified, and these genetic variations were assumed to influence the constitutive expression or the pregnane X receptor mediated induction of *CYP2C9*^12^. As a consequence of *CYP2C9* polymorphisms, substantial reduction in oral clearance of many clinically important drugs, such as *S*-warfarin, fluvastatin, gliclazide or glimepiride, celecoxib, phenytoin or valproate, has been reported that requires dose adjustment to avoid serious adverse reactions^4,5,11^. In patients with loss-of-function CYP2C9*2 or CYP2C9*3 alleles, reduced ability to metabolize the anticoagulant *S*-warfarin and therefore increased risk of over-anticoagulation are well-established^13,14^. *CYP2C9* genotype-guided dosing has been demonstrated to reduce bleeding complications during initiation of warfarin therapy^13,15,16^. In vitro studies with human liver microsomes and clinical studies involving patients with epilepsy have shown that carriers of CYP2C9*2 and/or CYP2C9*3 alleles resulted in decreased valproate metabolism compared to individuals with CYP2C9*1/*1 wild-type genotypes^17–19^. Although the association between *CYP2C9* genotype and valproate blood concentrations was clearly demonstrated in children whose metabolic pathways of valproate other than CYP2C9-mediated routes are poorly developed, the impact of *CYP2C9* genetic variability on valproate clearance was not significant in adult patients^20^.

Investigation of clinically relevant *CYP2C9* polymorphisms with decreased enzyme function is important in the interpretation of altered efficacy and/or toxicity of CYP2C9 substrate drugs, particularly of those with narrow therapeutic ranges^21^. Applying *CYP2C9* genotype testing in clinical practice may contribute to better understanding of phenotypic effect and thus to avoiding adverse events and/or impaired drug-response. Several clinical studies have been implemented to apply *CYP2C9* genotype-based therapies; however, inter-individual variability in CYP2C9 activity can be partly attributed to genetic polymorphisms. Non-genetic factors (age, hormonal status, disease, co-medication, nutrition) can further modulate CYP2C9 expression and CYP2C9 activity resulting in phenoconversion^6,22^. Inhibition of CYP2C9 activity or transcriptional induction of *CYP2C9* gene due to co-medication and other non-genetic factors can transiently switch into poor or extensive metabolizer phenotype. The *CYP2C9* promoter region contains several nuclear receptor responsive elements (e.g. for pregnane X receptor, constitutive androstane receptor, glucocorticoid receptor) that are involved in transcriptional regulation of CYP2C9 expression^23,24^. Considering both the genetic and non-genetic variations may be required for an accurate estimation of CYP2C9-mediated drug metabolism. The present study attempted to investigate the impact of the two most common *CYP2C9* alleles (CYP2C9*2 and CYP2C9*3) on CYP2C9 activity in liver tissue samples from adult organ donors belonging to Hungarian (Caucasian) population. The hepatic microsomal CYP2C9 activity was characterized by using tolbutamide as the CYP2C9-selective probe substrate. Since a minor role of CYP2C8 in tolbutamide 4′-hydroxylation has been suggested^25^, the impact of the clinically most relevant, loss-of-function CYP2C8*3 (rs11572080) on tolbutamide 4′-hydroxylase activity of tissue donors was also investigated. Furthermore, we aimed to identify some non-genetic factors (demographic parameters, co-medication) that can potentially modify the expression or the activity of CYP2C9. Incorporating these factors in prediction of CYP2C9 activity may improve *CYP2C9* genotype-based therapy.

## Material and methods

### Human liver microsomes and RNA samples

Human liver tissues (N = 164) were obtained from organ transplant donors at the Department of Transplantation and Surgery, Semmelweis University (Budapest, Hungary). The present study was approved by the Hungarian Committee of Science and Research Ethics, Medical Research Council (125/PI/2011, 4799-0/2011EKU) (https://ett.aeek.hu/en/secretariat/ access date: 08 June, 2021). The study was performed in accordance with the relevant guidelines and regulations (Act CLIV of 1997 on Health, decree 23/2002 of the Minister of Health of Hungary and the declaration of Helsinki). Written informed consent for CYP testing of liver tissues was obtained from transplant recipients. Clinical histories of the tissue donors are shown in Table 1. Human livers were perfused with Euro-Collin’s solution (Fresenius AG, Bad Homburg vdH, Germany) and excised. For the isolation of microsomal fraction, the tissues were homogenized in 0.1 M Tris–HCl buffer (pH 7.4) containing 1 mM EDTA and 154 mM KCl, and differential centrifugation was performed as described by van der Hoeven and Coon^26^. Microsomal protein content was determined by the method of Lowry et al. with bovine serum albumin as the standard^27^. Total RNA was also extracted from approximately 50 mg of liver tissues homogenized in 1 ml of TRIzol reagent (Invitrogen, Carlsbad, CA) according to the manufacturer’s instructions. The RNA was precipitated by using ethanol and stored in 0.1% diethylpyrocarbonate contained ultra pure water at − 80 °C for further analyses.

**Table 1:** Demographic data of the human organ donors.

Demographic data			
Donor number			164
Age (year)^a^			46 (18; 74)
Gender	Male/female		87/77
Cause of death	Accident	Car/motor/bike accident	4
		Seizure induced cerebral injury	1
		Suicide	3
		Unknown cerebral injury	29
	Cerebral hemorrhage/hematoma	Ruptured cerebral aneurysm	5
		Epidural hematoma	1
		Intraventricular hemorrhage	8
		Subarachnoid hemorrhage	29
		Subdural hemorrhage	7
		Unknown cerebral hemorrhage	9
	Stroke	Ischemic stroke	11
		Hemorrhagic stroke	2
	Tumour		45
	Unknown		10
Medical history	Amoxicillin + clavulanic acid therapy		7
	Chronic alcohol consumption		10
	Medication with CYP2C9 inducer		14
	Medication with CYP2C9 inhibitor		3

^a^Median (min; max).

### CYP2C9 enzyme assay

The method of Miners and Birkett was followed to determine tolbutamide 4′-hydroxylation activity selective for CYP2C9^28^. The incubation mixture contained NADPH-generating system (1 mM NADP, 10 mM glucose 6-phosphate, 5 mM MgCl_2_ and 2 units/ml glucose 6-phosphate dehydrogenase), human liver microsomes (1 mg/ml) and tolbutamide (1 mM). After 20-min incubation, the reaction was terminated by ice-cold methanol and the incubation mixture was centrifuged for 10 min at 10,000 × *g*. High-performance liquid chromatographic analysis was performed according to published method^28^. CYP2C9 enzyme assay for each donor was performed in triplicate.

### *CYP2C* genotyping

Genomic DNA was isolated from liver samples by Quick-DNA™ Universal Kit (Zymo Research, Irvine, CA). Hydrolysis SNP analysis for CYP2C9*2, CYP2C9*3 and CYP2C8*3 was performed by polymerase chain reaction (PCR) with TaqMan probes (Metabion, Planegg/Steinkirchen, Germany) as previously reported^29^. Real-time PCR was carried out with 30 ng of genomic DNA by using Luminaris Color Probe qPCR Master Mix (Thermo Fisher Scientific, Waltham, MA).

### Analysis of CYP2C9 mRNA levels by quantitative real-time PCR

Total RNA (3 μg) was reverse-transcribed into single-stranded cDNA by using Maxima First Strand cDNA Synthesis Kit (Thermo Fisher Scientific, Wilmington, DE). The real-time PCR with human cDNA was performed by using KAPA Fast Probes Mastermix (KAPA Biosystems, Cape Town, South Africa) and TaqMan probes for CYP2C9 (BioSearch Technologies, Novato, CA). The quantity of the target RNA relative to that of the housekeeping gene glyceraldehyde 3-phosphate dehydrogenase (GAPDH) was determined. GAPDH expression is constant in all cells and independent from experimental conditions; therefore, its expression was set to 1, and CYP2C9 mRNA levels were normalized by GAPDH expression. The sequences of primers and probes used for the real-time PCR analyses of CYP2C9 and GAPDH expression were previously reported by Déri et al. (2020)^30^.

### Statistical analysis

For liver tissue donors (N = 164), *CYP2C9* and *CYP2C8* genotypes (for CYP2C9*2, CYP2C9*3 and CYP2C8*3) as well as hepatic activities (N = 144) and/or mRNA expression (N = 109) of CYP2C9 were determined. Linkage disequilibrium between *CYP2C* SNPs was calculated using Haploview (v4.2; Broad Institute, Cambridge, MA)^31^. The frequency distribution of CYP2C9 activities were determined in 144 subjects, and three categories (low, intermediate, high) were distinguished for poor, intermediate and extensive metabolizers. The comparison of CYP2C9 enzyme activities or mRNA levels between various *CYP2C9* genotype groups was performed by Kruskal–Wallis ANOVA followed by Dunn’s multiple comparisons test (GraphPad Instat v3.05; GraphPad Software, San Diego, CA). The frequencies of CYP2C9 activity reducing factors were compared in subjects carrying CYP2C9*1/*1, and the differences between high intermediate/extensive metabolizers and low intermediate/poor metabolizer subjects were calculated by Fisher’s exact test. A 2-tailed *P*-value < 0.05 was considered to be statistically significant.

## Results

### *CYP2C9* genotypes of liver tissue donors

The two loss-of-function *CYP2C9* alleles most common in Caucasian populations (CYP2C9*2: 3608C > T, rs1799853 and CYP2C9*3: 42614A > C, rs1057910) were identified in liver tissue donors (N = 164). The wild-type CYP2C9*1 allele was assigned in the absence of CYP2C9*2 and CYP2C9*3. The genetic linkage between CYP2C9*2 and CYP2C8*3 is well-characterized^32^; therefore, the loss-of-function CYP2C8*3 (2130G > A, rs11572080; 30411A > G, rs10509681) allele was also identified in the tissue donors. The relative allele frequencies of CYP2C9*2, CYP2C9*3 and CYP2C8*3 alleles were found to be similar to those in the Caucasian populations (Table 2)^6,33^. The majority (approximately two thirds) of organ donors carried CYP2C9*1/*1 genotype, possessing the potential for having functional CYP2C9 enzyme. More than one fourth of tissue donors were heterozygous; 24 subjects carried CYP2C9*1/*2 and 21 displayed CYP2C9*1/*3 genotype. Six subjects carried mutations (SNPs) associated with decreased CYP2C9 activity in both alleles (CYP2C9*2/*2 or CYP2C9*2/*3). CYP2C9*3/*3 genotype was not detected in the investigated population. The SNPs in CYP2C9*2 and CYP2C8*3 alleles were in significant linkage (D’ 0.87; LOD 18.8) in tissue donors all belonging to the Caucasian population, whereas other SNP pairs were in linkage disequilibrium. Most subjects with CYP2C9*1/*2 were heterozygous for CYP2C8*3 (with CYP2C8*1/*3 genotype, 19/24), whereas of those with CYP2C9*1/*3, none of the subjects carried CYP2C8*3. Of the two tissue donors with homozygous for CYP2C9*2, one was also homozygous for CYP2C8*3; however, the other carried CYP2C8*1/*3 genotype. Furthermore, of those subjects carrying the combination of both *CYP2C9* alleles (CYP2C9*2/*3), two were heterozygous for CYP2C8*3 (CYP2C8*1/*3), and the other two were with homozygous wild genotype (CYP2C8*1/*1). Of 113 subjects having homozygous wild genotype (CYP2C9*1/*1), only two subjects were CYP2C8*1/*3 carrier.

**Table 2:** Allele and genotype frequencies of CYP2C9*2, CYP2C9*3 and CYP2C8*3 in liver tissue donors and in Caucasian population.

	N	Frequency (%)	
		Tissue donors	Caucasian population^a^
***CYP2C9* allele**			
*2	32	9.76	8–19
*3	25	7.62	3–16
***CYP2C8* allele**			
*3	26	7.9	6–14
***CYP2C9* genotype**			
*1/*1	113	68.9	55.3–61.9
*1/*2	24	14.6	11.8–28.2
*1/*3	21	12.8	8.5–25.7
*2/*3	4	2.4	0.9–8.9
*2/*2	2	1.2	0.5–8.5
*3/*3	0	0.0	0.0–5.7
***CYP2C8* genotype**			
*1/*1	139	84.8	74.3–81.7
*1/*3	24	14.6	17.6–24.0
*3/*3	1	0.6	0.7–1.7

^a^Allele frequencies in Caucasian population according to Zanger and Schwab^6^, Genotype frequencies in Caucasian population according to Zhou et al.^5^, Yasar et al.^32^, Dai et al.^71^, Takahashi and Echizen^72^, Scordo et al.^73^.

### Hepatic CYP2C9 activities

**Figure 1:**
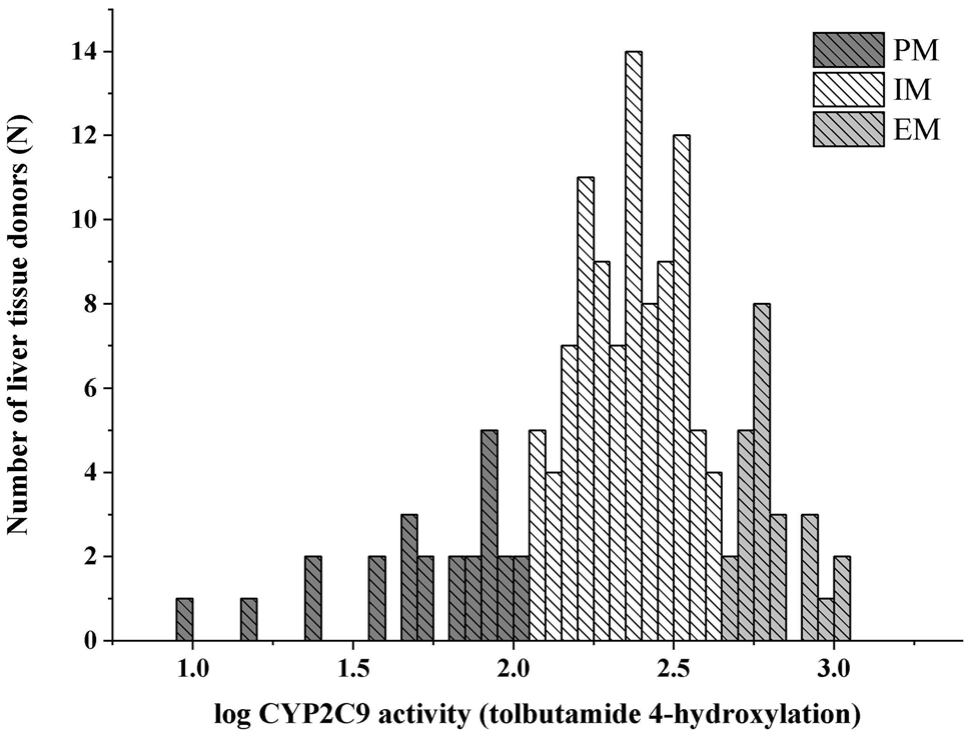
Frequency distribution of hepatic CYP2C9 activities (tolbutamide 4′-hydroxylation) in human tissue donors (N = 144). *PM* poor metabolizer, *IM* intermediate metabolizer, *EM* extensive metabolizer.

CYP2C9 enzyme activity was characterized in microsomal fractions from 144 human liver tissues. Tolbutamide was used as the probe substrate for CYP2C9, and the formation of 4′-hydroxy-tolbutamide was quantified based on “per mg microsomal protein per minute”. Variations of tolbutamide 4′-hydroxylation ranged from extremely low to rather high values displaying skewed distribution and more than two orders of magnitude differences between the lowest and the highest activities (9.47–1056 pmol*mg^−1^*min^−1^) (Fig. 1). On the basis of CYP2C9 activities, the liver tissue donors were classified into poor, intermediate and extensive metabolizer phenotype categories, and the cutoff values between the categories were 110 and 490 pmol*mg^−1^*min^−1^. In the intermediate metabolizer group, high and low intermediate metabolizer phenotypes were distinguished by the cutoff value of the median CYP2C9 activity (240 pmol*mg^−1^*min^−1^). The majority of liver tissues (N = 97) showed intermediate CYP2C9 activity, while 23 of the 144 were poor and 24 were classified as extensive metabolizers. No associations were found between CYP2C9 activities and the tissue donors’ demographic parameters, such as age and sex (*P* > 0.05, data not shown).

### Impact of *CYP2C9* genotype and non-genetic factors on hepatic CYP2C9 activities

For prediction of CYP2C9 activity and CYP2C9 phenotype from *CYP2C9* genotyping data, the basic assignments of poor-intermediate-extensive metabolizer phenotypes for various *CYP2C9* diplotypes were applied. Refinement of the genotype-to-phenotype classifications by Mostafa et al. and activity scoring recommended by Clinical Pharmacogenetic Implementation Consortium were also considered (Table 3)^34,35^. Subjects with two functional wild-type alleles were predicted to be normal metabolizers with extensive and high intermediate CYP2C9 activity. Those carrying CYP2C9*1/*2 were distinguished from those with CYP2C9*1/*3 displaying high intermediate and intermediate metabolizer phenotypes, respectively. While those subjects with two reduced-function alleles (CYP2C9*2/*2, CYP2C9*2/*3) were considered to be poor metabolizers (Table 3). However, the *CYP2C9* genotype-based phenotype prediction was not always consistent with the categories based on hepatic tolbutamide 4′-hydroxylation activity. Therefore, in addition to *CYP2C9* genotype, the impact of CYP2C8*3 allele and non-genetic phenoconverting factors (sex, medication, alcohol consumption) on CYP2C9 activity was investigated^22,32,36,37^. The impact of CYP2C9 inducers (dexamethasone, methylprednisolone, midazolam) and CYP2C9 inhibitors (amlodipine, tamoxifen) as well as of non-specific non-genetic factors, such as chronic alcohol consumption and amoxicillin + clavulanic acid therapy that can increase transcriptional expression of CYP2C9 or decrease CYP2C9 enzyme activities ^3,37–41^ was taken into account in CYP2C9 phenotype prediction (Table 3). The cohort of tissue donors were divided into four groups according to their *CYP2C9* genotypes (CYP2C9*1/*1, CYP2C9*1/*2, CYP2C9*1/*3 and CYP2C9*2/*2 or CYP2C9*2/*3). Subjects carrying CYP2C9*2/*2 and CYP2C9*2/*3 were grouped, because of the low number of tissue donors, and because these genotypes were predicted to have similar effect on CYP2C9 activity.

**Table 3:** Genotype-based prediction of CYP2C9 metabolizer phenotypes and phenoconversion by non-genetic factors.

*CYP2C9* genotype	Phenotype predicted from genotype		Phenoconversion		
	Classification according to Mostafa et al.^34^	Activity scoring according to CPIC guideline^35^	Medication with CYP2C9 inducer^a^	Medication with CYP2C9 inhibitor^b^	Non-specific non-genetic factors^c^
*1/*1	Normal (high IM–EM)	2	EM	IM–PM	IM–PM
*1/*2	High IM	1.5	EM	IM–PM	IM–PM
*1/*3	IM	1	High IM–EM	PM	PM
*2/*2	PM	1	IM	PM	PM
*2/*3	PM	0.5	IM	PM	PM
*3/*3	PM	0	IM	PM	PM

*PM* poor metabolizer, *IM* intermediate metabolizer, *EM* extensive metabolizer, *CPIC* Clinical Pharmacogenetics Implementation Consortium.

^a^CYP2C9 inducers: dexamethasone, methylprednisolone, midazolam.

^b^CYP2C9 inhibitors: amlodipine, tamoxifen.

^c^Non-specific factors: chronic alcohol consumption, amoxicillin + clavulanic acid therapy.

**Figure 2:**
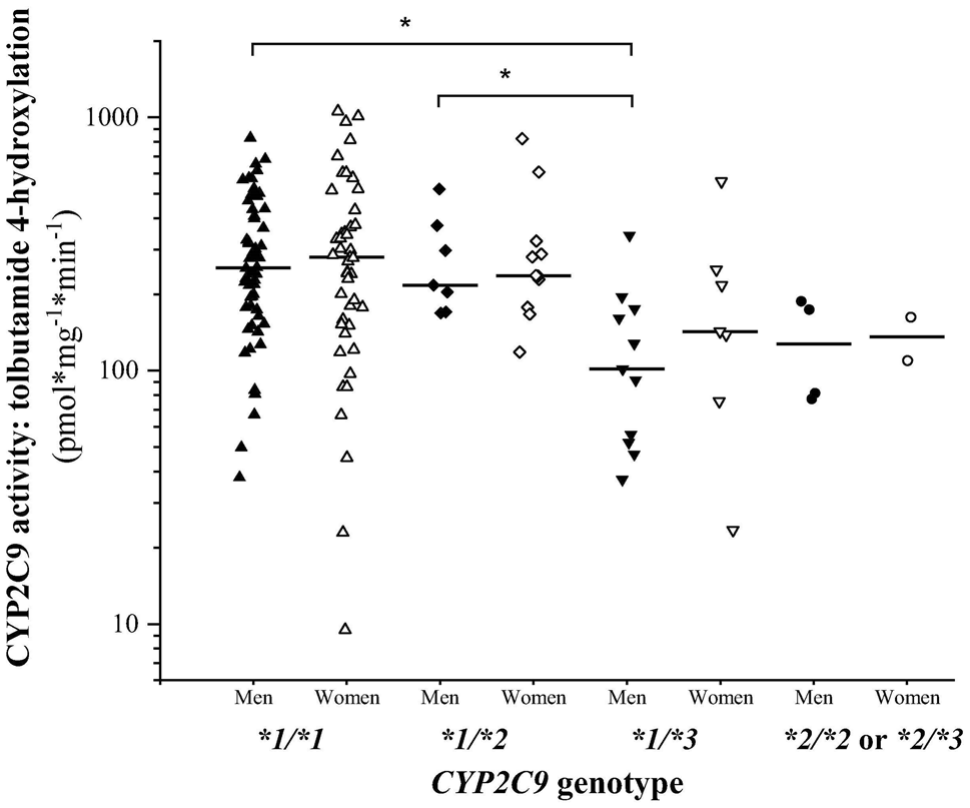
Gender-based differences in tolbutamide 4′-hydroxylation between various *CYP2C9* genotype groups. *Significant difference (*P* < 0.05).

Although in various *CYP2C9* genotype groups, no gender-based differences in tolbutamide 4′-hydroxylation were demonstrated, male subjects carrying CYP2C9*1/*3 displayed significantly lower activities than those males with CYP2C9*1/*1 or CYP2C9*1/*2 genotypes (CYP2C9*1/*1, CYP2C9*1/*2, CYP2C9*1/*3 and CYP2C9*2/*2 or *2/*3: 303.7 ± 180.78, 279.1 ± 129.96, 126.1 ± 90.26 and 130.0 ± 59.02 pmol mg^−1^ min^−1^; Kruskal–Wallis ChiSq = 15.9, N = 76, *P* = 0.0012) (Fig. 2). In female tissue donors, however, similar differences between various genotype groups were not observed (CYP2C9*1/*1, CYP2C9*1/*2, CYP2C9*1/*3 and CYP2C9*2/*2 or *2/*3: 332.4 ± 251.99, 310.8 ± 202.79, 201.0 ± 175.71 and 135.7 ± 37.45 pmol mg^−1^ min^−1^; Kruskal–Wallis ChiSq = 5.01, N = 68, *P* > 0.05). Therefore, gender-independent evaluation of phenoconversion was applied for other non-genetic factors.

**Figure 3:**
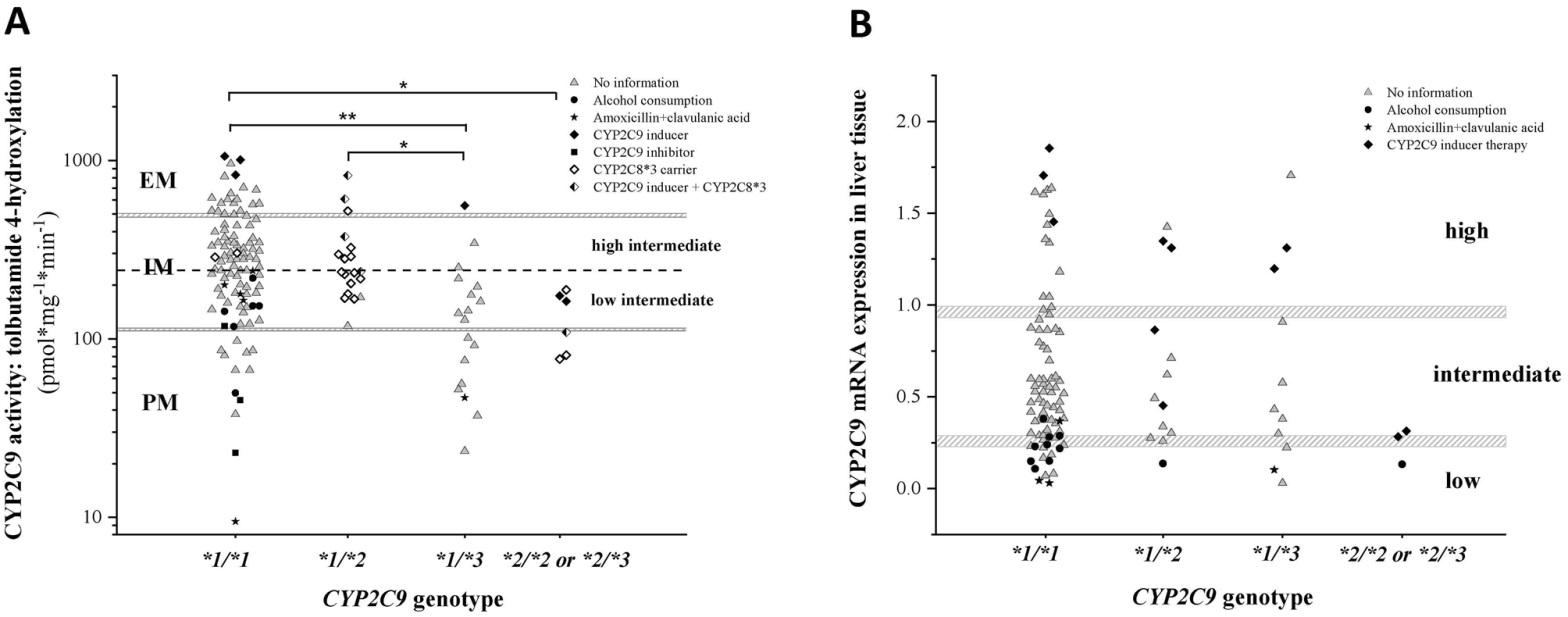
Hepatic CYP2C9 activity (tolbutamide 4′-hydroxylation) (**A**) and CYP2C9 expression (**B**) in subjects carrying various *CYP2C9* genotypes. CYP2C8*3 carriers and non-genetic factors (CYP2C9 inducer and inhibitor therapy, amoxicillin + clavulanic acid treatment, chronic alcohol consumption) found in clinical reports of the tissue donors are indicated. The median CYP2C9 activity (dotted line) is for the cutoff value between high and low intermediate metabolizers. *PM* poor metabolizer, *IM* intermediate metabolizer, *EM* extensive metabolizer, *low* low expression, *intermediate* intermediate expression, *high* high expression. *P < 0.05; **P < 0.001.

Most of the subjects with CYP2C9*1/*1 genotype predicting normal (high intermediate/extensive) CYP2C9 activity (Table 3) displayed intermediate (68.3%), extensive (19.8%) and poor (11.9%) tolbutamide 4′-hydroxylation (317.0 ± 216.19 pmol*mg^−1^*min^−1^) (Fig. 3A and Table 4). Of the 20 extensive metabolizers, 3 were treated with CYP2C9 inducers (dexamethasone, midazolam) that confirmed rather high CYP2C9 activity. Fourty three tissue donors, despite their homozygous wild genotype, displayed lower tolbutamide 4′-hydroxylase activity than predicted from the genotype, and were thus categorized as low intermediate and poor metabolizers. It was clearly recognized that the activity reducing non-genetic factors in the medical history of the CYP2C9*1/*1 carrier subjects were associated with low intermediate and poor CYP2C9 activities (14/43 in low intermediate/poor metabolizers *vs* 0/58 in high intermediate/extensive metabolizers, OR: 57.5, 95%CI: 3.31–998.61, *P* < 0.0001) (Fig. 3A). Fourteen of these low intermediate and poor metabolizer subjects carrying CYP2C9*1/*1 genotype were reported non-genetic factors in the medical history (amoxicillin + clavulanic acid therapy for 5, CYP2C9 inhibitor therapy for 3 and chronic alcohol consumption for 6) that explained the low tolbutamide 4′-hydroxylase activities. However, in the medical history of 29 tissue donors, there was no relevant information on the non-genetic factors responsible for low CYP2C9 activity, which means that the low tolbutamide 4′-hydroxylation activity of 28.7% of the CYP2C9*1/*1 carriers (29/101) could be explained by neither the polymorphic *CYP2C9* alleles investigated nor by the non-genetic phenoconverting factors reported in the medical history, and there was still a gap between *CYP2C9* genotype and phenotype. Significant differences in hepatic tolbutamide 4′-hydroxylation activity were observed between the subjects carrying CYP2C9*1/*1 or CYP2C9*1/*2 and CYP2C9*1/*3 (317.0 ± 216.19 or 299.1 ± 176.10 *vs* 155.3 ± 130.77 pmol*mg^−1^*min^−1^, Kruskal–Wallis ChiSq = 20.3, *P* = 0.0008). Although high intermediate metabolizer phenotype was predicted from CYP2C9*1/*2 genotype (Table 3), tolbutamide 4′-hydroxylation activity ranged from intermediate to extensive metabolism (Table 4). Of the 19 subjects with CYP2C9*1/*2 genotype, 4 tissue donors (2 extensive and 2 intermediate metabolizers) received CYP2C9 inducer drugs, whereas the medical history of 10 with low intermediate tolbutamide 4′-hydroxylation did not indicate any non-genetic factors that potentially decreased CYP2C9 activity (Fig. 3A). The subjects carrying CYP2C9*1/*3 (N = 18) were predicted to be intermediate metabolizers (Table 3); the CYP2C9 activities, however, extended wide range from poor to extensive metabolism (Table 4). The one extensive metabolizer subject with CYP2C9*1/*3 genotype was on CYP2C9 inducer therapy that clearly explained the relatively high activity. For one further CYP2C9*1/*3 carrier subject, amoxicillin + clavulanic acid therapy was reported in the medical history that might have resulted in low CYP2C9 activity. However, for the other 7 heterozygous subjects with CYP2C9*1/*3, there was no relevant information available that could explain the low CYP2C9 activities. For 45.9% of the tissue donors with one wild type and one polymorphic alleles (CYP2C9*1/*2 and CYP2C9*1/*3) (17/37), neither the *CYP2C9* genotype nor the non-genetic factors reported in the medical history explained the lower CYP2C9 activity than predicted from the genotype. The tissue donors with two reduced function alleles (CYP2C9*2/*2 and CYP2C9*2/*3) predicting poor metabolizer phenotype (Table 3) displayed poor-intermediate CYP2C9 activities that significantly differed from the activity of the CYP2C9*1/*1 genotype group (317.0 ± 216.19 *vs* 131.9 ± 48.78 pmol*mg^−1^*min^−1^, *P* = 0.0395). For 3 of them, CYP2C9 inducer therapy explained the elevated activities, whereas for 1 subject (16.6%), *CYP2C9* genotype and non-genetic phenoconverting factors could not confirm the intermediate activity. In conclusion, the *CYP2C9* genotype-predicted tolbutamide 4′-hydroxylation activity (Table 3) was false in approximately half of the tissue donor subjects (68/144), and non-genetic phenoconverting factors improved the activity prediction merely to 67.4% (false in 47 of 144, 32.6%). However, the CYP2C9 genotype–phenotype mismatch still existed in 32.6% of the subjects.

**Table 4:** Comparison of phenotype predictions based on *CYP2C9* genotype and hepatic CYP2C9 activity (N = 144) or CYP2C9 expression (N = 109) in tissue donors.

*CYP2C9* genotype	N	CYP2C9 phenotype based on hepatic CYP2C9 activity			CYP2C9 activity^a^
		PM	IM	EM	(pmol*mg^−1^*min^−1^)
*1/*1	101	12	69	20	278 (9.47; 1056)
*1/*2	19	0	16	3	237 (118; 824)
*1/*3	18	8	9	1	133 (23; 558)^b,c^
*2/*2 or *2/*3	6	3	3	0	136 (77; 188)^b^

		CYP2C9 phenotype based on hepatic CYP2C9 expression			
		PM	IM	EM	
*1/*1	82	14	50	18	0.502 (0.030; 1.853)
*1/*2	13	1	9	3	0.493 (0.137; 1.424)
*1/*3	11	3	5	3	0.432 (0.030; 1.705)
*2/*2 or *2/*3	3	1	2	0	0.208 (0.132; 0.283)

^a^Median (min, max).

^b^Significant difference from CYP2C9*1/*1.

^c^Significant difference from CYP2C9*1/*2.

Although it is known that CYP2C8 is able to metabolise several of CYP2C9 substrates to some extent, it is not clear whether *CYP2C8* polymorphism can contribute to the poor metabolism of tolbutamide; therefore, the effect of CYP2C8*3 on 4′-hydroxylation activity was also considered. The majority of liver tissue donors with CYP2C9*1/*2 genotype (17/19) carried the CYP2C8*3 allele, only 2 subjects with the lowest CYP2C9 activities of the CYP2C9*1/*2 group carried homozygous wild CYP2C8*1/*1 genotype. Furthermore, on the basis of tolbutamide 4′-hydroxylation activity, those subjects carrying CYP2C8*3 allele in CYP2C9*1/*1 genotype group were categorized to be high intermediate metabolizer. Of the subjects with two reduced-function alleles (CYP2C9*2/*2 and CYP2C9*2/*3), 4 carried CYP2C8*3 allele; however, they were phenotypic intermediate and poor metabolizers. From these results, CYP2C8*3 appeared to have no impact on hepatic tolbutamide 4′-hydroxylation.

### Association between *CYP2C9* genotype and hepatic CYP2C9 expression

In the present work, it was also investigated whether *CYP2C9* genotype was associated with hepatic CYP2C9 mRNA levels in 109 tissue donors (Fig. 3B and Table 4). In the evaluation, we considered non-genetic factors in the medical history of liver tissue donors that may affect CYP2C9 expression (CYP2C9 inducer therapy, chronic alcohol consumption, amoxicillin + clavulanic acid therapy). Most of the 109 subjects (75.2%) carried CYP2C9*1/*1 genotype. Of these homozygous wild CYP2C9*1/*1 genotype subjects, 17% expressed CYP2C9 mRNA at low, 61% at intermediate and 22% at high levels. The hepatic CYP2C9 expression of the heterozygous subjects carrying either CYP2C9*1/*2 or CYP2C9*1/*3, and of those with two polymorphic alleles (CYP2C9*2/*2 and CYP2C9*2/*3) did not differ from each other or from those of CYP2C9*1/*1 subjects (CYP2C9*1/*1, CYP2C9*1/*2, CYP2C9*1/*3 and CYP2C9*2/*2 or *2/*3: 0.628 ± 0.451, 0.657 ± 0.448, 0.651 ± 0.551 and 0.243 ± 0.098; Kruskal–Wallis ChiSq = 3.31, N = 109, *P* > 0.05). The non-specific non-genetic factors, such as chronic alcohol consumption and amoxicillin + clavulanic acid therapy were often demonstrated to be the culprit of the low CYP2C9 expression independently from *CYP2C9* genotype (for 12 subjects with CYP2C9*1/*1 genotype, for 1 with CYP2C9*1/*2, for 1 with CYP2C9*1/*3 genotype and for 1 with CYP2C9*2/*3 genotype) (Fig. 3B). Furthermore, CYP2C9 inducer therapy substantially increased CYP2C9 expression often evoking high CYP2C9 mRNA levels (for 3 subjects with CYP2C9*1/*1 genotype, for 2 with CYP2C9*1/*2 and for 2 with CYP2C9*1/*3 genotype). As a consequence of CYP2C9 inducer therapy, intermediate expression was observed in 2 additional subjects with CYP2C9*1/*2 genotype and in 2 subjects with two loss-of-function CYP2C9 alleles (CYP2C9*2/*2 and CYP2C9*2/*3).

## Discussion

CYP2C9 is in the focus of pharmacogenetic studies for genotype-based drug therapy, because it is one of the most abundant hepatic CYP enzymes and catalyses the metabolism of many clinically important drugs, particularly of those with narrow therapeutic concentration range^4,5,35^. The association between the clearance of the anticoagulant *S*-warfarin and *CYP2C9* polymorphism was the most intensively investigated, and to avoid bleeding complications, genotype-guided dosing was recommended^13–15,42^. Although no significant difference in in vitro losartan metabolism was found between CYP2C9*1/*2 and CYP2C9*1/*1 genotypes, a substantial decrease was detected in hepatic microsomes from subjects with CYP2C9*1/*3, CYP2C9*2/*2, and CYP2C9*3/*3 genotypes comparing to those with the homozygous wild-type genotype^43^. Similarly, no or negligible reduction of flurbiprofen metabolism was observed in healthy volunteers carrying CYP2C9*1/*2, whereas the metabolic rates of flurbiprofen were markedly decreased in subjects with CYP2C9*1/*3 or with two polymorphic alleles (CYP2C9*2/*2, CYP2C9*2/*3, CYP2C9*3/*3) comparing to those carrying two wild-type alleles^44^. Genetic variability of *CYP2C9*, however, can partly explain the substantial inter-individual variations in pharmacokinetics of CYP2C9 substrates. Due to phenoconversion, non-genetic factors can significantly modify CYP2C9 expression and/or enzyme activity predicted from the genotype^36,45^. The aim of the present work was to elucidate the impact of *CYP2C9* polymorphisms common in Caucasian populations on the activity and expression of CYP2C9 in liver tissues from 164 Hungarian organ donors. CYP2C9 enzyme activity was characterized by tolbutamide 4′-hydroxylation in microsomal fractions isolated from the liver tissues. Since a minor role of CYP2C8 enzyme in the hydroxylation of tolbutamide was suggested^25^, the contribution of the most common CYP2C8*3 allele to the reduction of tolbutamide 4′-hydroxylation was also investigated.

The allele frequencies for CYP2C9*2, CYP2C9*3 and CYP2C8*3 in the 164 tissue donors were similar to the allele frequency data previously reported in Caucasian populations^4–6,33^. The significant linkage between the CYP2C9*2 and CYP2C8*3 alleles (D′ = 0.87) demonstrated in the liver tissue donors was concordant with previous findings^32,46,47^. Since several CYP2C9 substrates (montelukast, fluoxetine, ibuprofen, rosiglitazone, tolbutamide, zopiclone) are also metabolized to some extent by CYP2C8^48^, reduced activity of CYP2C8*3 has been assumed to contribute to the low metabolic rates of CYP2C9 substrates in CYP2C9*2 carriers. A substantial decrease in ibuprofen clearance and as a consequence, increased risk of gastrointestinal bleeding have been reported in heterozygous CYP2C9*1/*2 subjects when CYP2C9*2 was present in combination with CYP2C8*3^46,49,50^. In vitro studies on diclofenac metabolism identified CYP2C9 as the major catalyst with minor contribution of CYP2C8^51^; however, the association between diclofenac clearance and genetic polymorphisms of *CYP2C9* and *CYP2C8* appear to be controversial^52,53^. The impact of *CYP2C9* genetic polymorphisms on tolbutamide clearance has clearly been demonstrated^54^; however, no data available for the *CYP2C8* polymorphic alleles. Although both CYP2C9*2 and CYP2C9*3 alleles are associated with decreased enzyme activity^55,56^, in heterozygous subjects carrying one wild-type and one polymorphic alleles (CYP2C9*1/*2 or CYP2C9*1/*3), the role of the CYP2C9*2 in modification of tolbutamide 4′-hydroxylase activity was found to be negligible in the liver tissues, whereas CYP2C9*3 was associated with significant reduction of CYP2C9 activity comparing to those with CYP2C9*1/*1 genotype. Furthermore, tolbutamide 4′-hydroxylase activity of the liver tissue donors with two loss-of-function alleles (CYP2C9*2/*2, CYP2C9*2/*3) was lower than in those with CYP2C9*1/*1. Our data are consistent with those of Kirchheiner et al.^54^ and Jetter et al.^57^, who demonstrated tolbutamide clearance in CYP2C9*1/*2 carriers to be indistinguishable from the subjects with two wild-type alleles, whereas the oral plasma clearance was significantly reduced in CYP2C9*1/*3 heterozygotes and in subjects with two polymorphic alleles comparing with those carrying CYP2C9*1/*1. In the present work, we have demonstrated for the first time that the presence of the loss-of-function CYP2C8*3 had no impact on tolbutamide 4′-hydroxylation in CYP2C9*1/*2 carriers. Even more, CYP2C8*3 in two of the CYP2C9*1/*1 carriers did not result in significant reduction of tolbutamide 4′-hydroxylase activity, but both subjects were high intermediate metabolizers.

In subjects with homozygous wild-type genotype (CYP2C9*1/*1), hepatic CYP2C9 mRNA expression has been reported to correlate with CYP2C9 activity^29^. However, the polymorphic CYP2C9*2 and CYP2C9*3 alleles seemed to have no impact on CYP2C9 expression. No significant differences in CYP2C9 mRNA levels were found between the subjects with CYP2C9*1/*1 genotype and those carrying one or two polymorphic *CYP2C9* alleles. The C > T transition at 3608 position in CYP2C9*2 allele and the nucleotide change of 42614A > C in CYP2C9*3 are well-described to result in amino acid changes that significantly impair CYP2C9 activity; however, these SNPs seem to have no impact on CYP2C9 mRNA expression^4^. Our findings confirmed that the CYP2C9*2 and CYP2C9*3 polymorphic alleles did not influence the hepatic CYP2C9 mRNA expression. It also means that in those carrying polymorphic *CYP2C9* alleles, CYP2C9 expression does not inform about hepatic CYP2C9 activity, whereas CYP2C9 mRNA levels correlate with CYP2C9 activity merely in those subjects with CYP2C9*1/*1 genotype, in line with previous findings^29^.

Because of the risk of serious adverse reactions, Clinical Pharmacogenetic Implementation Consortium has recommended *CYP2C9* genotype-based activity scoring to clinicians when prescribing CYP2C9 substrate drugs, such as non-steroidal anti-inflammatory agents, anticoagulant warfarin or anticonvulsant phenytoin^35,58,59^; however, internal and environmental non-genetic factors have also been suggested to consider. Due to phenoconversion, the concomitant treatments with CYP2C9 inhibitors and inducers have been proposed to improve CYP2C9 phenotype prediction. As a consequence of CYP2C9 inhibitor therapy, an individual with CYP2C9*1/*1 genotype can transiently become low intermediate or poor metabolizer, while the poor-metabolizer status of subjects with two polymorphic alleles has been assumed to be not affected by inhibitors^34^. In liver tissue donors carrying CYP2C9*1/*1 genotype, medication with CYP2C9 inhibitors (amlodipine, tamoxifen) was associated with low tolbutamide 4′-hydroxylation activity; furthermore, amoxicillin + clavulanic acid therapy and chronic alcohol consumption resulted in a decrease in CYP2C9 activity and mRNA levels. The racemic mixture of the anti-hypertensive amlodipine is used for therapeutic purposes; however, vasodilation is ascribed only to its *S*-enantiomer. Amlodipine has been reported to inhibit CYP2C9 activity in a stereoselective manner, and *R*-enantiomer was proved to be more potent CYP2C9 inhibitor than *S*-amlodipine^41^. The risk of drug interactions with CYP2C9 substrates has also been predicted during co-administration of the selective estrogen receptor modulator tamoxifen^60^. Tamoxifen and its anti-estrogenic hydroxylated metabolites (4-hydroxy-tamoxifen, endoxifen and norendoxifen) potently inhibited the activities of CYP2C enzymes. These findings were confirmed by the reduced tolbutamide 4′-hydroxylation activities in hepatic microsomes of tissue donors treated with amlodipine or tamoxifen. The pathomechanism of chronic alcohol consumption induced liver disease has long been studied, and there is a large body of evidence indicating that impaired drug metabolism is related to severe liver disease^61–63^. Amoxicillin, the widely used antibiotic is often applied in combination with clavulanic acid. Hepatotoxic effect of this combination is generally mild; however, amoxicillin + clavulanic acid therapy rarely leads to drug-induced liver injury or severe acute liver failure, for which liver transplantation is the only life-saving intervention^64,65^. Although both chronic alcohol consumption and amoxicillin + clavulanic acid therapy have been reported to exert liver injury, information about their CYP2C9 inhibitory potential is hardly available. In contrast to amlodipine and tamoxifen that have the capability to inhibit CYP2C9 function, chronic alcohol consumption and amoxicillin + clavulanic acid therapy evoking liver injury were likely to have non-specific impact on CYP enzyme function rather than direct CYP2C9 inhibitory properties^66,67^.

CYP2C9 inducers have also been reported to modify CYP2C9 metabolic activity; therefore, concomitant treatment with CYP2C9 inducer drugs is recommended to take into account during phenotype prediction^34^. As a consequence of inducer therapy such as rifampicin, dexamethasone, carbamazepine or phenobarbitone, higher CYP2C9 activity is expected in patients with one or two wild-type *CYP2C9* alleles than predicted from the genotype, whereas CYP2C9 function is assumed to be unchanged in those subjects with two polymorphic alleles^34^. The promoter region of the *CYP2C9* gene contains a number of nuclear receptor binding sites through which the gene can be transcriptionally induced. The synthetic glucocorticoid dexamethasone or the corticosteroid methylprednisolone are known to activate glucocorticoid receptor and pregnane X receptor, and to increase CYP2C9 transcription, whereas midazolam has been reported to act as a pregnane X receptor activator and to induce the expression of *CYP2C9*^3,38–40,68,69^. The liver tissue donors known to receive dexamethasone, methylprednisolone or midazolam displayed high CYP2C9 mRNA expression and activity. In those subjects carrying one or two wild-type alleles, the high tolbutamide 4′-hydroxylation activity and CYP2C9 mRNA level were associated with CYP2C9 inducer drug therapy, resulting in extensive or high intermediate metabolizer phenotypes. However, in those with two polymorphic alleles (CYP2C9*2/*2 or CYP2C9*2/*3), the effect of CYP2C9 inducer therapy on increasing CYP2C9 expression and function was observed, which did not confirm the phenoconversion prediction by Mostafa et al.^34^. Since both CYP2C9*2 and CYP2C9*3 display some residual activity, it is reasonable to assume that the presence of CYP2C9 inducers ameliorated the poor function of CYP2C9 predicted from genotype. In vitro studies with various *CYP2C9* allelic variants demonstrated that the inducibility of CYP2C9*2 and CYP2C9*3 by rifampicin was similar to that of CYP2C9*1^12^. As a result of rifampin treatment (pregnane X receptor activator), an increase in tolbutamide clearance (generally twofold) was reported in healthy volunteers with various *CYP2C9* genotypes, and the CYP2C9 inducibility by rifampin was observed in all genotype groups, even in CYP2C9*2/*2, CYP2C9*3/*3 or CYP2C9*2/*3 carriers^70^.

Some limitations of the present study should be considered. First, CYP2C9*3/*3 genotype was not detected in the present population, although the prevalence of this genotype was reported to be 0–5.7% in Caucasian populations^5,32,71–73^. Definite conclusion nevertheless could be drawn from CYP2C9*1/*3 genotype regarding the impact of CYP2C9*3 allele on the enzyme activity and the expression of CYP2C9. Second, we did not assess *CYP2C9* alleles other than CYP2C9*2 and CYP2C9*3; however, the prevalence of other clinically relevant *CYP2C9* alleles in Caucasian populations are extremely low^74^. Third, one may assume that the medical history of some subjects was incompletely reported, and some relevant non-genetic factors that can decrease or increase CYP2C9 activity were not included.

In conclusion, the impact of *CYP2C9* polymorphic alleles (CYP2C9*2, CYP2C9*3) and non-genetic factors on CYP2C9 function and mRNA expression was demonstrated in human liver tissues. The role of the CYP2C9*3 allele in functional impairment was clearly confirmed, whereas the influence of CYP2C9*2 allele on hepatic tolbutamide 4′-hydroxylation activity was evident in those subjects carrying two polymorphic alleles. Furthermore, the contribution of CYP2C8*3 to tolbutamide 4′-hydroxylase activity was not confirmed. Although *CYP2C9* genotype was found to be a major factor in CYP2C9 function, non-genetic factors such as co-medication with CYP2C9 inhibitors and inducers as well as non-specific factors including amoxicillin + clavulanic acid therapy and chronic alcohol consumption significantly altered the CYP2C9 phenotype predicted from genotype. In more than two thirds of the liver tissue donors, the combined effect of the *CYP2C9* genotype and non-genetic factors was found to correspond to CYP2C9 function. It should be emphasized that non-genetic factors affected tolbutamide 4′-hydroxylation activity of CYP2C9 both in CYP2C9*1/*1 carriers and in those subjects with one or two polymorphic alleles. In contrast, substantial variability in hepatic CYP2C9 mRNA levels, irrespective of the *CYP2C9* genotype, was demonstrated; however, CYP2C9 induction and non-specific non-genetic factors potentially resulting in liver injury appeared to contribute to CYP2C9 expression. As such these results supported the complex implementation of *CYP2C9* genotype and non-genetic factors for the most accurate estimation of hepatic CYP2C9 enzyme activity that can improve efficiency and safety of medication with CYP2C9 substrate drugs in clinical practice. However, a CYP2C9 genotype–phenotype mismatch still existed in 32.6% of the subjects.

## Abbreviations

CYP: Cytochrome P450
GAPDH: Glyceraldehyde 3-phosphate dehydrogenase
PCR: Polymerase chain reaction
SNP: Single nucleotide polymorphism
